# Editorial: Emerging Mechanisms in Purinergic Signaling: From Cell Biology to Therapeutic Perspectives

**DOI:** 10.3389/fphar.2020.01022

**Published:** 2020-07-07

**Authors:** Rosa Gomez-Villafuertes, Elena Adinolfi

**Affiliations:** ^1^ Departamento de Bioquímica y Biología Molecular, Facultad de Veterinaria, Universidad Complutense de Madrid, Madrid, Spain; ^2^ Instituto Universitario de Investigación en Neuroquímica, Universidad Complutense de Madrid, Madrid, Spain; ^3^ Instituto de Investigación Sanitaria del Hospital Clínico San Carlos, Madrid, Spain; ^4^ Section of Experimental Medicine, Department of Medical Sciences, University of Ferrara, Ferrara, Italy

**Keywords:** adenosine, ATP, purinergic receptors, purinergic signaling, ectonucleotidases

Purinergic signaling or purinome is the common name for a complex ensemble of receptors, extracellular enzymes, and transporters interacting with extracellular ATP and its degradation products ADP, AMP, and adenosine. It was thanks to the early discovery of professor Burnstock that ATP, previously tough to exert its function manly intracellularly, could also activate extracellular signals and to his strong support to the existence of a non-adrenergic non-cholinergic neurotransmission that purinergic signaling took its first steps. Subsequently, many studies proved that the purinome is involved not only in neurotransmission but also in the activation of the immune response, carcinogenesis and the etiopathology of several conditions including but not limited to neuropathic and inflammatory pain, neurological disorders, cardiovascular, infectious, skeletal, reproductive, and immune system diseases, alterations of sense organs, airways, skin, muscles, gut, kidney and urinary tract. The purinome includes the P2X ATP-gated ion channels, metabotropic receptors for ATP (P2Y) and Adenosine (ADORA) but also ectonucleotidases such as CD39 and CD73 that are responsible for the hydrolysis of ATP to its derivatives ADP, AMP and adenosine. The intracellular pathways activated by this plethora of receptors include classical G-proteins coupled c-AMP and inositol triphosphate pathways, but also kinases and nuclear factors among which MAPK, Akt, PI3K, NF-kB, HIF-1α and NFAT. From a pharmacological point of view, several potent and selective agonists/antagonists, allosteric modulators, and blocking antibodies targeting most purinergic receptors and ectonucleotidases are available. Some of these compounds are currently used in therapy such as the antithrombotics targeting P2Y_12_ receptor, and multiple clinical trials are in progress to explore purinergic agents for the treatment of different neurological, inflammatory, and oncologic diseases among others. Nevertheless, a further research effort is required to better identify the right drugs or agonist/antagonist combinations and diseases to be targeted with purinome-centered therapies. This Research Topic aimed to gather new data and opinions on physio-pathological roles played by the purinome components.

Research articles in this topic include a brief report by Pegoraro et al. that investigates the effect of P2X7 receptor expression on HHV-6A infection, showing that P2X7 antagonism decreases viral load. Moreover, the authors demonstrate that P2X7 489C>T polymorphism correlates with HHV-6A infection in women affected with idiopathic infertility, a condition previously shown to correlate with HHV-6A infection. These data point to the P2X7 receptor as a potential therapeutic target to prevent HHV-6 infection and associated infertility.

Previous studies suggest that both ATP and adenosine consistently reduce the sinoatrial node spontaneous activity leading to negative cardiac chronotropy. Here, Bragança et al. demonstrate that activation of P2X4 ionotropic receptors plays a major role in decreasing the spontaneous activity of the sinoatrial node while partially offsetting the negative inotropic effect of the nucleotide in paced rat ventricles. These results strongly suggest that P2X4 agonists have the potential to became novel well-tolerated heart-rate lowering drugs with promising benefits in patients with deteriorated ventricular function.

In the field of neuroprotection, Alves et al. analyze the expression of P2Y receptors in the cortex following status epilepticus and determine the impact P2Y_1_ modulation on cortical damage using a unilateral mouse model of intra-amygdala kainic acid-induced status epilepticus. This study extends previous data and confirms anticonvulsive and neuroprotective properties of P2Y_1_ antagonism during status epilepticus, suggesting P2Y_1_-based treatment as possible new therapy for drug-resistant status epilepticus.

The ecto-5’-nucleotidase CD73 plays an important role in regulating vascular permeability and immune cell function. In this regard, Caiazzo et al. evaluate the effect of CD73 inhibition in the development of inflammation in the carrageenan-induced pleurisy model. This study demonstrates that inhibition of CD73 exacerbates the early phase of carrageenan-induced pleurisy by controlling pleural effusion and polymorphonuclear migration *in vivo* and *ex vivo*. On this basis, the authors suggest that CD73 might represent a valid biomarker for pleural effusion and a potential target for novel therapeutic interventions.

Another study included in the topic and covering lung inflammation is that of Santana et al., which investigates the role of the P2Y_12_ receptor in silicosis, demonstrating that inhibition of P2Y_12_ signaling with clopidogrel prevents silica-induced changes in lung function, and significantly reduces lung inflammation, fibrosis, as well as cytokine and nitrite production, thus preventing lung functional alterations and mortality.

Overviews by purinome experts are also present in this Research Topic including the article by Stokes et al. that summarizes the current evidence on the physiological roles of P2X receptors and discuss whether the use of pharmacological agents enhancing P2X receptor activity would offer a therapeutic benefit. Based on the advances in structural information and continued progress in allosteric binding pocket identification, plus access to the relevant animal models of disease, the authors convincingly suggest that positive modulation of P2X receptors will become a new fruitful area of research.

Corciulo and Cronstein give an overview of the purinergic system in the joint describing the expression and function of purinome components in the synovia, cartilage, ligament, tendon, and bone and highlighting the therapeutic perspective of targeting purinergic signaling in this anatomical area.

Similarly, Khalafalla et al. summarize the role of purinergic receptors in salivary gland function and dysfunction, analyzing their potential as therapeutic targets to promote saliva flow, prevent salivary gland inflammation, and enhance tissue regeneration.

In addition, Wei et al. review recent studies that support the role of Piezo1 channel as an intrinsic mechanosensor to trigger ATP release and subsequent purinergic receptor activation in several types of mechanosensitive cell types such as urothelial cells, endothelial cells, red blood cells, and mesenchymal stem cells.

In conclusion, we believe that the articles presented in this Research Topic cover multiple aspects of the ongoing research in the purinergic field and show the difficult task of fully-understanding the complexity of this ubiquitous signaling cascade.

Finally, we would like to dedicate the issue to the memory of Professor Geoffrey Burnstock, who recently passed away. He will always be an example of genius and passion for us and will be much missed by the scientific community. May he rest in peace and continue challenging dogmas wherever he is.

## Author Contributions

RG-V and EA have equally contributed to the work, and approved it for publication.

## Funding

We would like to thank all authors and reviewers for their valuable contribution. Also, we would like to acknowledge the support of MICINN (PID2019-109155RB-100), Universidad Complutense de Madrid (PR65/19-22453), Red de Excelencia Consolider-Ingenio Spanish Ion Channel Initiative (BFU2015-70067REDC), Italian Association for Cancer Research (AIRC IG 22837), and University of Ferrara.

## Conflict of Interest

The authors declare that the research was conducted in the absence of any commercial or financial relationships that could be construed as a potential conflict of interest.

